# Understanding the intention-behavior gap in brain decisions on green express packaging: An ERP study

**DOI:** 10.1371/journal.pone.0355783

**Published:** 2026-09-21

**Authors:** Zhenshui Yan, Tianbo Wang, Shuwei Wang, Wei Lyu

**Affiliations:** 1 School of Economics and Management, Shenyang Aerospace University, Shenyang, China; 2 School of Economics and Management, Anhui Polytechnic University, Wuhu, China; Instituto Superior de Contabilidade e Administracao de Lisboa - Instituto Politecnico de Lisboa (ISCAL-IPL), PORTUGAL

## Abstract

Accelerating the green transformation of express packaging is crucial for sustainable logistics. This study combines behavioral experiments with event-related potential (ERP) technology to explore the interactive effects of green advertising appeals and price premiums on consumers’ willingness to pay for green express packaging. Behavioral results reveal that price premium act as a primary structural barrier, with consumers’ green choices decreasing significantly as costs increase. The ERPs data uncover the underlying psychological conflict during decision-making: in the early stage, high price premium elicited larger N2 amplitudes, reflecting increased cognitive conflict. Notably, egoistic appeals triggered the most pronounced N2 conflict under high-cost conditions. In the late evaluative stage, altruistic appeals significantly enhanced LPP amplitudes under low-premium conditions, reflecting higher motivational significance and moral satisfaction. However, this effect was neutralized as costs crossed a psychological threshold. These findings suggest that green advertising’s regulatory potential is strictly anchored by economic costs. This study provides a neurophysiological perspective for optimizing pricing and marketing strategies in digital green logistics.

## Introduction

With the ubiquity of the internet and the rapid evolution of e-commerce, online shopping has become an indispensable component of daily life. By 2024, Chinese online shopping population had surged to 958 million, accounting for 85.3% of the total Internet user base [1]. Concurrently, as an inseparable pillar of the e-commerce ecosystem, the express delivery industry has continued to flourish, with total business volume surpassing 170 billion parcels in 2024, solidifying China’s position as one of the world’s largest logistics markets [2]. However, explosive growth in delivery volume has catalyzed a dramatic rise in the demand for packaging materials, leading to massive resource consumption [3]. The vast majority of domestic express packaging currently utilizes non-biodegradable materials [4]. These materials are not only difficult to recycle but are also predominantly incinerated alongside household waste, releasing harmful gases such as sulfur dioxide and nitrogen oxides, which poses significant threats to public health and exerts severe negative pressure on the environment [5]. Consequently, accelerating the green transformation of express packaging has become a strategic necessity for the sustainable development of the e-commerce industry.

To address these environmental challenges, the Chinese government has explicitly prioritized the development of green logistics. Notably, the “14th Five-Year Plan for Modern Logistics Development” emphasizes advancing the application of green packaging solutions [6]. Green packaging is defined as a sustainable form that maximizes resource conservation and minimizes environmental degradation throughout its entire life cycle [7]. As a critical subset of this broader concept, Green Express Packaging (GEP) is specifically engineered for the unique demands of logistics and delivery. Driven by these policy incentives, numerous enterprises have launched initiatives to integrate biodegradable or recyclable materials into their supply chains [8]. Similarly, e-commerce platforms have introduced strategies ranging from shared delivery boxes to source reduction packaging [9]. While these green packaging solutions have transitioned from conceptual designs to commercially viable products, a paradoxical gap remains: in the context of Chinese e-commerce platform landscape, mainstream platforms currently do not offer GEP as a selectable service option for consumers during the checkout process. This discrepancy suggests that the diffusion bottleneck of GEP cannot be resolved by top-down governance and corporate supply alone; rather, it hinges critically on active consumer engagement and their willingness to embrace these sustainable alternatives [10].

Despite the rising public awareness of environmental protection, the adoption of GEP often entails a price premium, requiring consumers to bear additional costs [11]. Previous literature suggests that consumers’ willingness to pay (WTP) is negatively correlated with the magnitude of this premium [12], a trend particularly pronounced among price-sensitive groups [13]. Specifically, regarding the Chinese market, empirical studies revealed a nuances landscape of consumers’ acceptance. Lin and Wang [14] found that residents in first-tier cities exhibit WTP of approximately ¥2.46 for GEP. Meanwhile, Jia et al. [15] reported a slightly lower but consistent WTP of ¥2.18 in second- and third-tier cities. Although these questionnaire-based studies indicate a general readiness to pay, actual green purchasing behavior remains infrequent [16]. This discrepancy highlights a critical methodological limitation—traditional behavioral data often reflect deliberate judgments formed during late-stageocesr cognitive psing [17], thereby failing to capture the pre-conscious, objective neural responses during the initial decision-making phase [18]. Against this backdrop, several critical questions remain unanswered: How do consumers genuinely evaluate GEP options when they are presented in a real-time e-commerce environment? More importantly, are there effective psychological interventions that can bridge the “willingness-to-action” gap and stimulate actual payment behavior? One promising solution lies in green advertising, which serve as a strategic tool for enterprises to communicate ecological values and product information to consumers [19]. Green advertising plays a pivotal role in guiding sustainable consumption and fostering corporate green development [20,21]. As the core of advertising messaging, advertising appeals—the specific persuasive strategies used to stimulate consumer desire—has a complex yet profound impact on purchase intentions [22]. While scholars have increasingly utilized green appeals emphasizing environmental attributes [23] to align with consumer values [24], their effectiveness in mitigating the psychological burden of GEP price premiums remains to be empirically tested from a neurocognitive perspective.

This paper aims to elucidate consumer WTP for GEP services within the e-commerce landscape, specifically focusing on the trade-offs between various price premiums and green advertising appeals. By combining behavioral experiments with Event-Related Potential (ERP) technology, the study captures both the explicit choice outcomes and the implicit neural underpinnings of the consumer decision-making process. While behavioral data identifies the critical price thresholds, the high-temporal-resolution ERPs analysis uncovers the cognitive conflicts and emotional processing that occur prior to the final decision. The findings offer empirical evidence to assist e-commerce platforms and logistics providers in optimizing GEP pricing strategies and advertising framing. Ultimately, this study contributes to the literature on sustainable consumption by providing a dual-layered perspective understanding of the behavioral and neurophysiological mechanisms required to bridge the gap in digital green logistics.

## Literature review and hypothesis development

### Green advertising appeals

Green advertising conceptualized as promotional activity centered on environmental protection and human health [23,25]. Within this domain, green advertising appeals serve as the strategic bridge between product attributes and consumer psychology. By creative presentation employed by businesses to articulate environmental benefits and value positions of a product, thereby fostering eco-friendly values and facilitating sustainable purchase behavior [26]. Crucially, the effectiveness of these appeals hinges on their matching degree with the consumers. When the style of an advertising appeal aligns appropriately with consumer psychology, it can explicitly establish a psychological connection with consumers, leading to heightened identification and emotional resonance [24,27]. Septianto et al. [28] and Prakash et al. [29] have categorized green advertising appeals into egoistic appeals and altruistic appeals. Egoistic appeals focus on the direct benefits to the consumer, such as personal health, safety, or economic savings; Altruistic appeals emphasize the broader benefits to others, the ecological environment, and society.

Existing research predominantly posits that consumers are inherently driven by self-interest; consequently, they are often more easily persuaded by egoistic advertising appeals. In addition, the efficacy of these appeals depends not only on the core message but also on how the information is processed by the consumer’s cognitive system. For instance, Li and Zhang [30] demonstrated that specific advertising copy can trigger consumer resonance, while the use of capitalized text has been found to significantly enhance attentional capture [31]. Furthermore, the visual aesthetics of the advertisement play a crucial role; cool-toned colors (e.g., blue and green) have a more positive impact on consumer emotions and purchase intentions compared to warm tones [32]. Beyond textual and color cues, the inclusion of environmental certification or recycling logos can remarkably strengthen pro-environmental behaviors [33].

### Dual-system theory and hypothesis development

Dual-system theory posits that human behavioral phenomena are the products of two distinct underlying psychological processes [34]. This framework suggests that human cognitive activity can be divided into two systems: System 1 operates as a heuristic-based mechanism characterized by rapid, unconscious, and emotionally driven information processing. In contrast, System 2 is a deliberate and self-controlled system that is relatively slow, conscious, and reliant on rational analysis [35]. While both systems operate concurrently, they contribute differently to problem-solving and decision-making [36]. To date, scholars have extensively applied dual-system theory within the domain of consumer behavior. In marketing, this framework has been used to explain purchase decision for healthy processed foods [37], analyze promotional decision-making [38] and predict impulsive purchase intentions [39]. Furthermore, in the field of e-commerce, Jiao et al. [40] employed this theory to explore the mechanisms underlying consumer loyalty.

In this study, the consumers’ choice of delivery method at the checkout stage can be conceptualized as a cognitive competition dominated by these two systems. Specifically, green advertising appeals, through vivid and resonant textual cues, are primarily designed to trigger the rapid, affective processing of System 1. This fosters an initial positive emotional inclination toward GEP through emotional resonance. Subsequently, the GEP price premium acts as an economic signal that demands rational weighing, thereby awakening System 2 for a more calculated evaluation. System 2 may integrate this additional cost and potentially suppress the emotional impulses initially generated by System 1.

In addition, product type likely plays a moderating role in this cognitive process. Following the classification by Nelson [41], When purchasing experience products (where attributes are required through experience), consumers’ cognitive orientations are naturally more aligned with the emotion-driven System 1, making them more susceptible to the influence of advertising appeals. Conversely, when evaluating search products (where attributes can be evaluated by prior information gathering), consumers tend to rely on the analytical and rational mode of System 2, rendering them more sensitive to price information. Consequently, the following hypotheses are proposed:

*H1.* Product type moderates the effect of green advertising appeals on consumers’ WTP for GEP.

### ERP method and ERP components

Previous research on green advertising and GEP price premiums has predominantly relied on self-reported questionnaires, which are susceptible to subjective bias and lack objective neurophysiological evidence. ERPs refer to the bioelectric signals generated by the nervous system in response to specific events (e.g., the presentation of a stimulus). By collecting and analyzing these signals, researchers can infer the complex temporal dynamics of the brain internal processing under particular stimulus [42]. Currently, ERP methodology has been extensively employed in marketing to explore the complex cognitive processes underlying price perception [43–45] and advertising effectiveness [46]; [47]. To reveal the physiological mechanisms of how advertising appeals and price premiums influence consumer decisions, this study integrates ERPs with behavioral experiments, focusing on two key components: N2 and LPP.

#### N2.

The N2 is a negative potential that generally appears in the frontal region of the brain, peaking between 200 and 350 ms. It serves as a neural index for conflict detection and information processing [48]. When consumers encounter information that conflicts with their internal preferences or expectations, larger N2 amplitudes are typically observed. For instance, differences in interface layouts on news webpages [49], monetary incentive strategies for online comments in e-commerce [50], and interplay between consumer privacy concerns and advertising transparency [51] have been found to elicit distinct N2 amplitudes. Based on dual-system theory, when consumers encounter a green advertisement followed by a GEP price premium, a cognitive conflict arises between the affective impulse to support the environment sustainability (driven by System 1) and the rational resistance to additional economic costs (driven by System 2). This transition between processing modes is expected to manifest in N2 variations. Consequently, the following hypothesis is proposed:

*H2.* The interaction between green advertising appeals and price premiums will induce significant N2 variations, reflecting the cognitive conflict during the evaluation of GEP services.

#### LPP.

The Late Positive Potential (LPP) is a positive-going component distributed across the central-parietal region, typically peaking between 600 and 1000 ms. In neuroscientific research, the LPP is a robust indicator of the evaluation process, whereby individuals who engage in a more thorough analysis of a stimulus tend to exhibit larger LPP amplitudes [52]. Specifically, LPP amplitudes reflect the allocation of attention and the motivational significance attributed to emotional visual stimulus [53,54]. Studies have demonstrated that enhanced LPP amplitudes are linked to the evaluative processing of both positive and negative emotional stimuli [53,55,56] with negative states often eliciting greater amplitudes than neural or positive ones [57]. Moreover, the LPP has been utilized in consumer neuroscience to optimize marketing strategies. For instance, Song et al [45] have employed LPP analysis to design innovative pricing mechanisms aimed at mitigating negative consumer emotions. Additionally, Li & Liu [58] have shown that adjusting the presentation order of production information on e-commerce platforms can modulate LPP amplitudes to stimulate positive purchasing emotions. In the present study, egoistic appeals that foster stronger emotional resonance are expected to interact with price signals and product types to modulate cognitive engagement, thereby manifesting in differentiated LPP amplitudes. Consequently, the following hypothesis is proposed:

*H3.* Green advertising appeals and price premiums will interactively influence the LPP amplitude, reflecting the late-stage motivational evaluation of GEP options.

## Study 1: Behavioral experiment

Study 1 aimed to examine consumers’ delivery preferences for GEP by simulating e-commerce checkout scenarios, specifically focusing on how product types and price premiums interact with green advertising appeals to influence decision-making. This study was approved by the Institute of Neuroscience and Cognitive Psychology of AHPU. All participants were informed about the study procedures and oral informed consent prior to participation.

### Participants and procedures

In the behavioral experiment, 900 participants were recruited through the Credamo platform. Upon enrollment, participants were randomly assigned to one of the eight experimental scenarios. After excluding samples with unusual short completion times or patterned response behaviors, a total of 819 valid samples (59.5% female) were retained for final analysis. The distribution of participants across the eight conditions was shown in Table 1.

**Table 1:** Experimental design and sample distribution.

Group	Product Type	GEP Advertising Appeals	GEP Price	N
1	search product	altruistic	2¥	104
2	search product	altruistic	4¥	100
3	search product	egoistic	2¥	106
4	search product	egoistic	4¥	101
5	experience product	altruistic	2¥	107
6	experience product	altruistic	4¥	101
7	experience product	egoistic	2¥	102
8	experience product	egoistic	4¥	98

### Stimulus materials

To maximize ecological validity, this experiment simulated the authentic checkout interface of Taobao, Chinese leading e-commerce platform, as the primary experimental stimulus. To ensure participants focused on the core decision-making variables, the interface was standardized and simplified by removing redundant information such as store names, while three essential areas were retained: product information, the green advertising banner, and the delivery selection panel. Consequently, a 2 (product type: search or experience) × 2 (green advertising appeal: egoistic or altruistic) × 2 (price premium: 2¥ or 4¥) three-factor mixed design was employed. Participants were randomly assigned to one of the eight experimental groups. Each participant viewed a specific checkout page stimulus and was asked to choose their preferred delivery method.

#### Product selection and pre-test.

To identify representative search and experience products, ten common consumer items were initially selected from Taobao. A pre-test was conducted with 45 participants who were first briefed about the definitions of search-based and experience-based products. Then they were asked to use a 7-point Likert scale to rate their agreement with the statement: Based on available online information, can this product be fully evaluated prior to purchase? 1 = completely evaluative before purchase, 7 = only evaluative after purchase [41,59]. Based on the results, power banks, mobile hard drives, Bluetooth earphones, wireless mice, and watches were categorized as search products, while perfume, hydrating masks, shoes, sunscreen, and toothpaste were categorized as experience products. To minimize confounding variables, we selected a power bank (M = 3.267, SD = 2.137) and a hydrating mask (M = 5.378, SD = 1.957) as the final stimuli. A paired t-test confirmed a significant difference between the two (t = −4.340, p < 0.001), ensuring a successful manipulation of product type.

#### Manipulation of advertising appeals and price.

**Fig 1:**
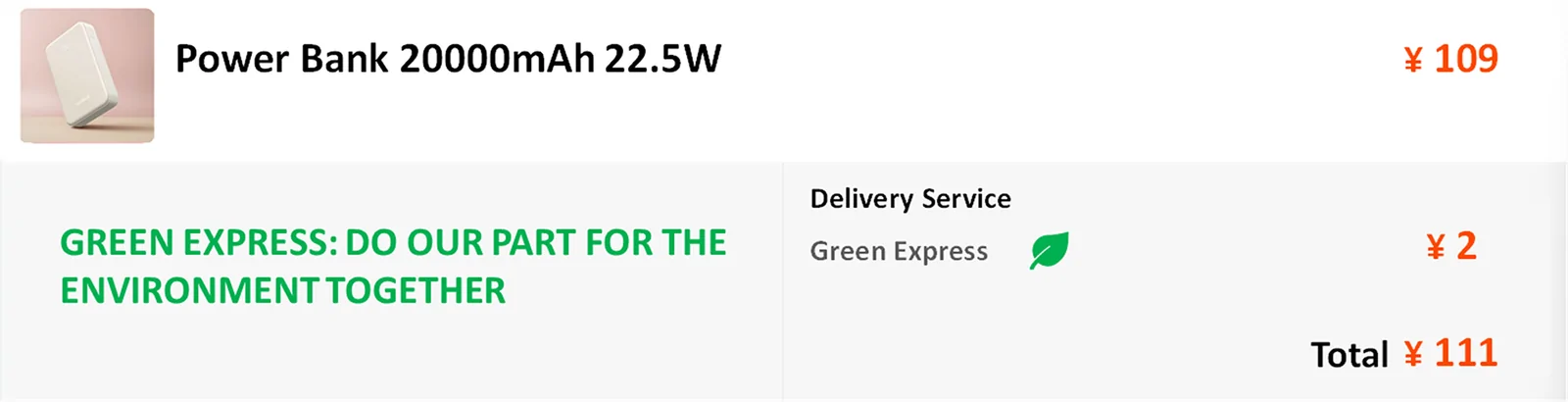
Example of the experimental stimulus.

Following the methodology of Kareklas et al. [60], 45 participants same with the previous text were asked to evaluated four advertising slogans (A1:Green Express, do our part for the environment together; A2:Green Express, reduce carbon footprint, lighten the earth’s load; A3:Green Express, add peace of mind to your personal health; A4: Green Express, show your eco-friendly attitude) by a 7-point Likert scale to rate their agreement with 2 dimensions: altruistic appeal (e.g., The advertisement is based on environmental protection, pro-social, or ecological balance considerations) and egoistic appeal (e.g., The advertisement is based on product value or personal interest considerations). All slogans were standardized in a light green color [32] and presented in capitalized fonts [31]. A manipulation check (as shown in Table 2) confirmed that participants clearly perceived the intended appeal types. To illustrate the experimental task, Fig 1 presents a sample of the simulated checkout interface used in study 1.

**Table 2:** Manipulation check of advertising appeals.

Advertisement	M (altruistic)	M (egoistic)	p	t	d
A1	6.133	3.4	<0.001	8.222	1.226
A2	6.267	2.867	<0.001	8.965	1.336
A3	3.345	5.778	<0.001	−6.489	0.967
A4	3.356	6.607	<0.001	−8.816	1.314

The price premiums for GEP were set at 2¥ and 4¥. These values were determined based on empirical evidence regarding Chinses consumers’ WTP for GEP. According to Jia et al. [15], while 74.1% consumers from second- or third-tier cities support GEP, their WTP is relatively low, typically not exceeding 5¥. Lin & Wang [14] suggested that the average WTP for GEP among Chinese consumers from first-tier city within the range 2.12¥ to 4.33¥. Therefore, 2¥ and 4¥ represent realistic low and high premium thresholds that align with current consumer psychological expectations and market feasibility.

### Results of Study 1

For the behavioral experiment, descriptive statistical analysis was initially conducted to examine the choice proportions for green express across the eight experimental conditions. The results indicated that the price premium served as the primary determinant of consumer decisions: the choice proportion for GEP was substantially higher at the 2¥ (M = 0.752) than at the 4¥ (M = 0.560). This price-dependent effect remained consistent across all combinations of advertising appeals and product types, as illustrated in Table 3.

**Table 3:** Choice proportion for GEP across experimental groups.

Group	Product Type	GEP Advertising Appeals	GEP Price	Green chioce %
1	search product	altruistic	2¥	0.692
2	search product	altruistic	4¥	0.550
3	search product	egoistic	2¥	0.802
4	search product	egoistic	4¥	0.515
5	experience product	altruistic	2¥	0.701
6	experience product	altruistic	4¥	0.554
7	experience product	egoistic	2¥	0.814
8	experience product	egoistic	4¥	0.633

To systematically assess the impact of product type, green advertising appeal, and price premium on consumer decisions, a binary logistic regression was performed using SPSS 27.0. In this model, the delivery choice served as the dependent variable, while product type, advertising appeal, price premium and their respective interactions were included as predictors. The Hosmer-Lemeshow test yielded a chi-square value of 0.806 (df = 6, p = 0.992), indicating a robust model fit. The regression analysis revealed a highly significant main effect of the price premium (B = −0.837, SE = 0.250, p < 0.001), with an odds ratio (Exp (B)) of 0.433, suggesting that for every unit increase in the price level, the likelihood of choosing GEP decreased by 56.7%. In contrast, the main effects of product type (B = 0.614, SE = 0.492, p = 0.213) and green advertising appeal (B = −0.703, SE = 0.670, p = 0.294) were not statistically significant, implying that variations in product category or advertising framing do not directly shift final payment behavior when immediate economic costs are present. Thus, H1 was not supported. Notably, a marginally significant interaction was observed between the price premium and green advertising appeal (B = 0.263, SE = 0.154, p = 0.087), hinting at the existence of differentiated cognitive processing pathways for green advertising appeals under varying levels of price pressure.

### Discussion of Study 1

Study 1 aims to identify the core drivers and inhibitors of consumer WTP for GEP. The regression results demonstrate that the price premium is the dominant structural barrier to green payment decisions. Regardless of the product type or the framing of green advertising appeals, the direct economic cost remains the primary determinant, suggesting that price sensitivity effectively overrides other psychological or contextual factors. Specifically, the non-significant main effect of product type indicates that consumers’ support for GEP dose not fluctuate based on the nature of the goods. Furthermore, the lack of a significant main effect for advertising appeals provides critical insights for green marketing: in the current experimental context, merely conveying environmental benefits to society or individuals is insufficient to construct a perceived value that compensates for an immediate financial sacrifice, which diverges from previous research suggesting that green advertising might enhances purchase intentions [61]. This reflects a discounting of green value propositions when weighed against tangible costs, further validating the green attitude and behavior gap in sustainable consumption [16].

However, these behavioral outcomes may mask the complex internal trade-offs occurring during the decision-making progress. Although the interaction between price and green advertising appeals was only marginally significant (p = 0.087), a nuanced observation of the descriptive data reveals that under low premium conditions, certain advertising appeals began to manifest higher choice proportions. This suggests that advertising framing may indeed trigger differentiated cognitive processing at an earlier stage, but such psychological inclinations are ultimately suppressed by the overwhelming rational calculation of cost-benefit in the final choice phase. This leads to a pivotal research question that behavioral data alone cannot resolve: Do different green advertising appeals induce distinct neurocognitive processing pathways despite the uniform behavioral output? It is possible that altruistic or egoistic message creates latent positive inclinations that are eventually defeated by the psychological conflict induced by the price premium. To reveal the cognitive mechanisms behind these behavioral results, Study 2 was introduced.

## Study 2: ERP experiment

Study 2 aims to explore the deep-level impact of green advertising appeals on the green decision-making progress and seek to capture the neurophysiological evidence of cognitive conflict and value evaluation that behavioral measures fail to detect.

### Participants

A total of 23 healthy college students were initially recruited for the study and the participants ranged in age from 22 to 30 years (M = 25.05, SD = 2.305). All participants were right-handed and possessed normal or corrected-to-normal vision. None of the individuals reported a history of serious physical, neurological, or psychiatric disorders. Furthermore, all participants had prior experience with online shopping on various e-commerce platforms. Before the commencement of the experiment, each participant provided written informed consent. The study protocol received formal approval from the Institute of Neuroscience and Cognitive Psychology of AHPU, all participants were informed about the study procedures prior to participation and each participant received a compensation of 40 RMB upon completion of the task. Due to excessive artifacts or individual reasons, data from four participants were excluded from the final analysis. Consequently, valid electroencephalogram data from 19 participants were utilized for the formal statistical processing.

### Materials and methods

A 2 (Green advertising appeal: altruistic or egoistic) ×2 (Price premium: 2¥ or 4¥) within-subjects repeated measures design was employed for the current ERP experiment. Although the analysis focused on these four conditions, the stimuli were presented in a sequence of three components labeled as S1, S2, and S3 to simulate a realistic online shopping process. Each stimulus of S1 consisted of a product image with its name text below to minimize potential habituation effects caused by excessive repetition. Based on the pre-test results from Study 1, power bank (M = 3.267, SD = 2.137) and mobile hard drives (M = 3.178, SD = 2.229) were selected as search products, while perfume (M = 5.200, SD = 1.878) and hydrating masks (M = 5.378, SD = 1.957) served as experience products. To eliminate confounding effects from physical attributes such as color or design, 6 presentative images (three for both product types) with similar market prices ranging from 80¥ to 100¥ were sourced from Taobao e-commerce platform. All S1 images were standardized to a resolution of 480 × 360 pixels to ensure visual consistency. S2 consisted of two types of GEP advertising slogans that validated in Study 1: altruistic appeals (A1 and A2) and egoistic appeals (A3 and A4). These slogans were presented in a light green color and capitalized fonts the same with Study 1. S3 presented a simulated interface requiring a choice between two delivery options: traditional express (free fee) and green express (2¥ or 4¥ the same as Study 1). In total, the stimulus set comprised 48 unique combinations (4 products pictures × 4 green advertising slogans × 2 price premium). Each combination was presented 5 times, resulting in a total of 240 experimental trials. This design ensured that each of the four core experimental conditions yielded 60 trials per participants, satisfying the requirements for an adequate signal-to-noise ratio in subsequent ERP component averaging.

### Procedures

The ERP experiment was divided into 6 blocks, with short breaks between them to prevent participants’ fatigue. Each block consisted of 40 stimuli combinations integrating S1, S2, and S3. All combinations were randomized. The presentation of all stimuli was controlled by the E-prime 3.0 software package (Psychology Software Tools, Pittsburgh, PA, United States) and stay 1 meter away from participants’ eyes.

**Fig 2:**
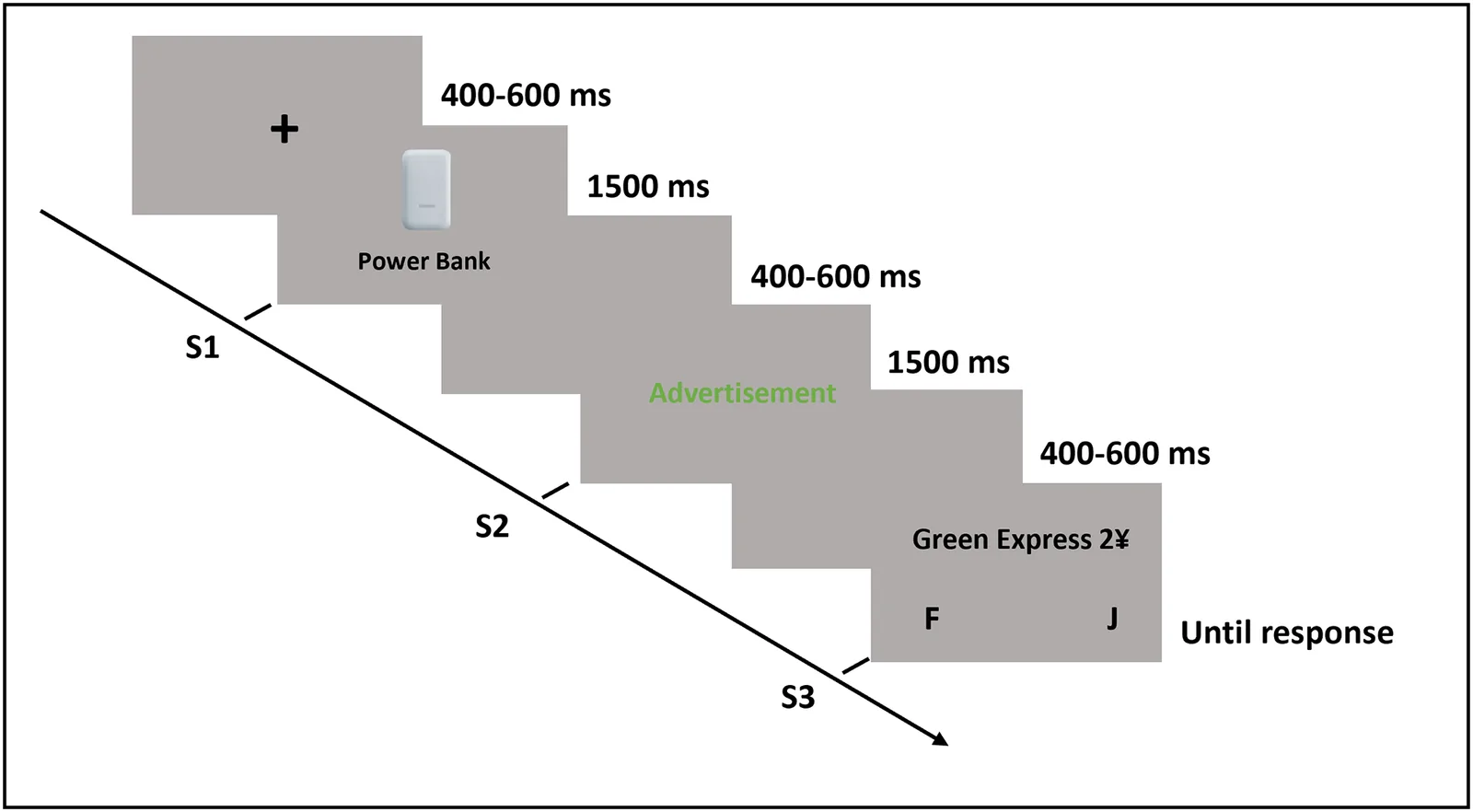
Experimental task: The participants were instructed to browse a product photo (S1) and the green express advertising appeals (S2) before making a final decision based on the price premium (S3).

The participants were seated in a sound-attenuated room and instructed to fix on the center of the computer screen. They were provided with the following instructions: “You are simulating the checkout process for products (S1) then viewing the green express advertising (S2), please make your express service choice (S3) after that.” Once participants confirmed their understanding of the experimental procedure, they could press SPACE key to initiate each trial. Each trial as shown in Fig 2, a red fixation cross “+” was presented against a gray background for 400–600 ms. Then, S1 was presented for 1500 ms, followed by a blank screen for 400–600ms, and S2 for 1500 ms. After another blank screen for 400–600ms, S3 appeared. Participants were instructed to make an express serve choice by press the F key for traditional express or the J key for green express. The S3 remained until a response was made. Then a new trial begins again (as shown in Fig 2). After the instructions, 8 training trials were provided to each participant as practice trials. Upon completion of the training trials, they could opt for additional practice or proceed to the formal trials. After the experiment concluded, each participant was compensated with 50¥ for their participation.

### Electroencephalography recording and analysis

Electroencephalography (EEG) data were recorded with a NeuroScan SynAmps2 Amplifier and the Curry 7.0 software (Neurosoft Labs, Inc., Sterling, VA, United States). A 32-channel Ag/AgCl electrode cap, positioned according to the international extended 10–20 system, was utilized for signal acquisition at a sampling rate of 1000 Hz. The EEG signals were referenced to the left mastoid and the ground electrode was placed at the prefrontal position. To monitor ocular activity, vertical and horizontal electrooculograms (EOG) were recorded from bipolar pairs of electrodes placed above and below the left eye and 10 mm lateral to the outer canthi of both eyes, respectively. All electrode impedances were maintained below 10 kΩ throughout the experiment.

Offline data processing was performed using Curry 7.0. The raw data were band-pass filtered between 0.1 and 30 Hz. Continuous EEG was segmented into epochs time-locked to the onset of the decision interface (S3), ranging from 200 ms pre-stimulus to 800 ms post-stimulus, with the 200 ms pre-stimulus interval serving as the baseline for correction. Artifacts such as blinks and eye movements were corrected, and any remaining trials with voltage fluctuations exceeding ±100 µV were automatically rejected. Finally, data were averaged into two conditions (2 Green advertising appeals × 2 Price premium).This procedure ensured that each participants retained at least 30valid trials per experimental condition, providing a robust signal to noise ratio for the target components [62].

### Results of Study2

**Fig 3:**
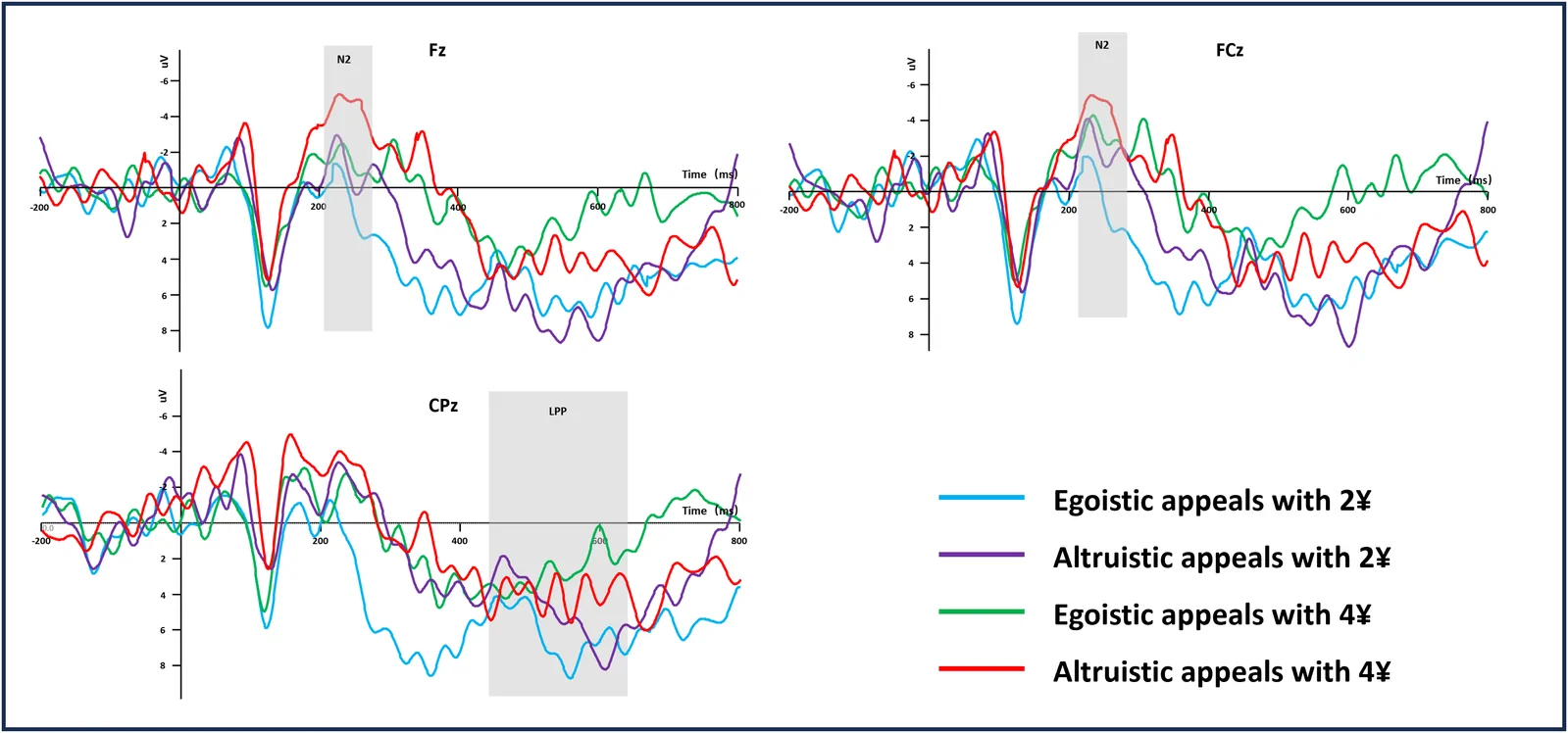
Grand-average ERPs waveforms for the N2 and LPP components at channels Fz, FCz and CPz.

Fig 3 illustrates the grand average ERP waveforms under the conditions of green advertising appeal types and GEP price levels. Repeated-measures analysis of variance was conducted on the mean amplitudes of the N2 and LPP components within two pre-specified time windows.

For the mean N2 amplitudes in the 210–280 ms window, a 2 (green advertising appeal: egoistic vs. altruistic) × 2 (price: 2 ¥ vs. 4 ¥) × 3 (electrode: Fz, FCz, Cz) three-way repeated-measures ANOVA was performed. The results revealed that the main effect of green advertising appeal was not statistically significant (p > 0.1). However, the main effect of price was significant (p < 0.001). Crucially, a significant interaction effect between advertising appeal and price was observed, F (1, 18) = 6.692, p = 0.019, η²ₚ = 0.271. Simple effect analysis indicated that under the 2 ¥ condition, no significant difference in N2 amplitudes was found between egoistic and altruistic appeals (p > 0.1). Conversely, under the 4 ¥ condition, a significant difference emerged, F (1, 18) = 6.296, p = 0.022, η²ₚ = 0.159. Specifically, egoistic appeals (M = −5.792, SE = 0.682) elicited significantly more negative-going N2 amplitudes compared to altruistic appeals (M = −4.612, SE = 0.553).

For the LPP component within the 600–800 ms window, a 2 (green advertising appeal: egoistic vs. altruistic) × 2 (price: 2 ¥ vs. 4 ¥) × 3 (Electrode: Cz, CPz, Pz) three-way repeated-measures ANOVA was conducted. While the main effect of advertising appeal was non-significant (p > 0.1), the main effect of price reached significance (p < 0.001). Furthermore, a significant interaction between advertising appeal and price was identified, F (1, 18) = 9.604, p = 0.006, η²ₚ = 0.348. Detailed simple effect analysis showed that in the 2 ¥ condition, altruistic appeals (M = 7.569, SE = 0.495) elicited significantly higher positive-going LPP amplitudes than egoistic appeals (M = 6.436, SE = 0.369). However, under the 4 ¥ condition, the mean LPP amplitude for altruistic appeals (M = 3.255, SE = 0.205) was significantly lower than that for egoistic appeals (M = 4.254, SE = 0.372).

### Discussion of Study2

While Study 1 revealed that the final choice of express delivery was primarily driven by price rather than product type or advertising appeal, the interaction between advertising and price was marginally significant. Study 2 employed N2 and LPP as core ERP components to delve into the subconscious evaluation process regarding green advertising and price premiums in e-commerce checkout scenarios, providing neurophysiological evidence for understanding green logistics consumption.

The ERP results indicated that a high price premium (4¥) elicited larger N2 amplitudes during the early stages of subconscious processing compared to a low premium (2¥). As a well-established index of cognitive conflict, the enhanced N2 amplitude reflects the brain’s detection of a discrepancy between the high cost and the individual’s internal expectations [63]. Notably, the egoistic appeal under the 4¥ condition triggered the most pronounced N2 conflict. This suggests that the incongruity between self-interest-oriented messaging and high real-world costs exacerbates early-stage psychological dissonance. Consequently, Hypothesis 2 is supported.

Furthermore, during the late conscious evaluation stage, low-price conditions induced significantly larger LPP amplitudes than high-price conditions. Since the LPP reflects the allocation of cognitive resources for emotional processing and regulation [64,65], this finding suggests that consumers mobilize more resources to mitigate the negative effect triggered by higher costs. In the 2¥ condition, altruistic appeals elicited the highest LPP amplitudes, indicating that when economic barriers are within an acceptable range, pro-social motivations can effectively activate positive evaluative resources and enhance the perceived value of green consumption. However, under the 4¥ condition, the LPP amplitude for altruistic appeals decreased significantly, falling below that of the egoistic group. This suggests that excessive economic costs not only neutralize the emotional dividends of altruistic appeals but may also trigger psychological reactance, such as a sense of moral coercion. Accordingly, Hypothesis 3 is supported. Taken together, the N2 and LPP results offer a comprehensive understanding of the cognitive trajectory from early conflict monitoring to late value appraisal in consumers’ evaluation of green delivery options.

## General discussion

### Research finding

This study investigates the internal neural processing mechanisms underlying consumers’ decisions regarding express delivery options within e-commerce checkout scenarios by combining behavioral experiments with event-related potentials.

The behavioral results from Study 1 reveal a forbidding reality that while green consumption is widely advocated by society, price remains the core barrier to green choices in price-sensitive e-commerce express contexts, which aligns with the research of previous scholars [16]. When the price premium doubles from 2¥ to 4¥, consumers’ green selection behavior experiences a significant decline. Such a trend confirms that the gap between green consumption intentions and actual behavior truly exists in the process of logistics greening [14,15]. Although product types and green advertising appeals did not exert a significant main effect on final payment behavior, the discovery of a marginally significant interaction between advertising appeal and price suggests the regulatory potential of green appeals in the early stages of cognitive processing.

Study 2 further explores the essence of this interaction through ERP technology at the neural level. The results show that as a monitoring index of cognitive conflict [48], the high premium of 4¥ elicited stronger N2 amplitudes during the early stages of cognitive processing compared to the 2¥ condition. This effectively captures the psychological struggle individuals face between environmental moral pursuits and the perception of financial loss. Specifically, egoistic advertising appeals induced the greatest N2 conflict under the high-price condition, suggesting a strong resistance in the brain toward the discrepancy between self-benefit messaging and disproportionately high costs.

In the late stage of decision-making, the LPP component reveals the allocation mechanism of emotional resources. Under low-premium conditions, altruistic advertising appeals can effectively evoke a sense of moral satisfaction and soothe the initial psychological conflict caused by price. However, once the premium crosses a certain psychological threshold, the LPP amplitude for altruistic appeals decreases significantly. This suggests that excessive costs not only neutralize previous positive emotions but also allow feelings of moral coercion or financial anxiety to supersede environmental enthusiasm.

### Theoretical contributions and practical implications

This study extends the existing literature on sustainable consumption in several significant ways. First, by integrating dual-system theory with green consumption, the research provides a quantitative characterization of the temporal interplay between System 1, which represents intuitive emotion, and System 2, which embodies rational logic. This approach not only supplements traditional green decision-making models such as the theory of panned behavior [66] but also offers a novel neurological perspective to explain the underlying mechanisms of green choices.

Second, this research challenges the conventional assumption regarding the universal effectiveness of advertising appeals in promoting green consumption [67]. The findings demonstrate that the regulatory effect of such appeals is strictly anchored by economic costs, revealing the boundary conditions under which green marketing can influence consumer psychology. Finally, the study confirms the stability of green delivery decisions across both search and experience products. This indicates that the willingness to pay for green packaging is primarily driven by the characteristics of the logistics process itself rather than the type of commodity purchased. Such a finding helps define the research boundaries for green logistics within the broader e-commerce environment.

Based on the aforementioned findings, this study offers actionable strategic recommendations for promoting the green transformation of the express delivery industry. Considering the intense psychological conflict triggered by higher price premiums, the results validate previous questionnaire-based findings regarding the thresholds of price acceptability in express delivery [14,15]. To minimize the cognitive conflict detected by the N2 component in the consumer brain, e-commerce platforms and logistics companies should ideally maintain the green packaging premium within a 2¥ limit.

In addition, when implementing green marketing for express packaging, firms should adopt a differentiated communication strategy based on cost structures. Under low-cost conditions, altruistic green advertising copy should be promoted to evoke positive social emotions. However, when logistics costs remain elevated, marketers should not emphasize environmental emotional appeals and instead highlight features beneficial to personal interests, such as the superior physical protection and durability of the packaging, to avoid triggering consumer resistance.

### Limitations and future research

Despite providing a robust cognitive model to explain the neural dynamics of GEP decisions, this study has several limitations for future exploration. First, the participant sample was primarily composed of young college students. While this demographic represents a significant portion of active e-commerce users, their price sensitivity and environmental attitudes may differ from those of the broader population. Future research should incorporate a more diverse social sample to examine how individual differences, such as income levels, educational background, and preexisting environmental consciousness, moderate the neural response to green price premiums. Second, this research focused specifically on the static decision-making process at the moment of e-commerce checkout. In reality, sustainable logistics is a continuous process that extends beyond the point of purchase. Future studies could investigate consumer attitudes and behaviors regarding the reuse and recycling of green packaging after delivery, potentially exploring how the physical experience of the product influences long-term brand loyalty and subsequent green choices. Third, as digital technology continues to evolve, future research could leverage virtual reality and augmented reality environments to explore how enhanced presence and immersion alter individual perceptions of green premiums.
